# Comparative physiological and transcriptomic analyses reveal ascorbate and glutathione coregulation of cadmium toxicity resistance in wheat genotypes

**DOI:** 10.1186/s12870-021-03225-w

**Published:** 2021-10-08

**Authors:** Tao Zhang, Jingui Xiao, Yongsheng Zhao, Yifan Zhang, Yaqi Jie, Dandan Shen, Caipeng Yue, Jinyong Huang, Yingpeng Hua, Ting Zhou

**Affiliations:** 1 School of Life Sciences, Zhengzhou University, Zhengzhou, 450001 People’s Republic of China; 2 School of Agricultural Sciences, Zhengzhou University, Zhengzhou, 450001 People’s Republic of China

**Keywords:** Wheat, Cd resistance, Genotypic diversity, ROS, AsA-GSH cycle

## Abstract

**Background:**

Cadmium (Cd) is a heavy metal with high toxicity that severely inhibits wheat growth and development. Cd easily accumulates in wheat kernels and enters the human food chain. Genetic variation in the resistance to Cd toxicity found in wheat genotypes emphasizes the complex response architecture. Understanding the Cd resistance mechanisms is crucial for combating Cd phytotoxicity and meeting the increasing daily food demand.

**Results:**

Using two wheat genotypes (Cd resistant and sensitive genotypes T207 and S276, respectively) with differing root growth responses to Cd, we conducted comparative physiological and transcriptomic analyses and exogenous application tests to evaluate Cd detoxification mechanisms. S276 accumulated more H_2_O_2_, O_2_^−^, and MDA than T207 under Cd toxicity. Catalase activity and levels of ascorbic acid (AsA) and glutathione (GSH) were greater, whereas superoxide dismutase (SOD) and peroxidase (POD) activities were lower in T207 than in S276. Transcriptomic analysis showed that the expression of *RBOHA*, *RBOHC*, and *RBOHE* was significantly increased under Cd toxicity, and two-thirds (22 genes) of the differentially expressed *RBOH* genes had higher expression levels in S276 than inT207. Cd toxicity reshaped the transcriptional profiling of the genes involving the AsA-GSH cycle, and a larger proportion (74.25%) of the corresponding differentially expressed genes showed higher expression in T207 than S276. The combined exogenous application of AsA and GSH alleviated Cd toxicity by scavenging excess ROS and coordinately promoting root length and branching, especially in S276.

**Conclusions:**

The results indicated that the ROS homeostasis plays a key role in differential Cd resistance in wheat genotypes, and the AsA-GSH cycle fundamentally and vigorously influences wheat defense against Cd toxicity, providing insight into the physiological and transcriptional mechanisms underlying Cd detoxification.

**Supplementary Information:**

The online version contains supplementary material available at 10.1186/s12870-021-03225-w.

## Background

Cadmium (Cd) is a heavy metal that is harmful to animals, plants, and microorganisms. With progressing industrialization and urbanization, Cd pollution is becoming increasingly serious in arable land and irrigation water, where it is absorbed by plants. Cd is highly mobile in soil; thus, it is easily absorbed by plants, resulting in retarded plant growth. Further, Cd is highly mobile in plants and easily contaminates agricultural products via vascular transportation [1]. It subsequently enters the human body via the food chain, and excessive intake or inhalation of Cd can damage the human immune, urinary, bone, nervous, reproductive, and other systems [2]. In addition, Cd has strong carcinogenic, teratogenic, and mutagenic effects [3, 4], resulting in diseases, such as “Itai-Itai disease” [5]. Hence, Cd has been listed as one of the 12 hazardous substances worldwide with environmental and food safety significance by the United Nations Environment Program since 1984 [6].

Cd toxicity inhibits seed germination and root growth, reduces photosynthesis, blocks the xylem, induces stomatal closure, and causes water and nutrient imbalances in plants [7, 8]. Besides, it stimulates the burst of reactive oxygen species (ROS), and the excessive ROS causes the loss of cellular membrane integrity and damages the molecular structure of DNA, proteins, and lipids [1, 9]. As a main and direct site for Cd adsorption and perception, the root has evolved various strategies to cope with Cd toxicity. These include the firm fixation of Cd in the root cell wall, reduced Cd transport to the aboveground parts, the expulsion of Cd from the root into the soil, or transportation of Cd into non-metabolic organelle vacuoles [10, 11]. Another important resistance mechanism relies on enhancing antioxidant defense. Two ways are used to reduce oxidative damage and help maintain redox homeostasis in plants [12]: 1) the enzymatic antioxidant scavenging system that includes superoxide dismutase (SOD), peroxidase (POD), catalase (CAT), ascorbate peroxidase (APX), glutathione-S-transferase (GST), monodehydroascorbate reductase (MDHAR), and glutathione reductase (GR); and 2) the non-enzymatic antioxidants system, including ascorbic acid (AsA), glutathione (GSH), and nicotinamide adenine dinucleotide phosphate (NADPH) [13]. Among these antioxidants, reduced compounds play key roles because peroxidases can react with H_2_O_2_ rapidly, and their reduced forms are quickly regenerated by specific reductases [14]. AsA and GSH have strong reducibility and are localized in almost all cellular compartments, including the mitochondria, chloroplasts, peroxisomes, nuclei, cytosol, endoplasmic reticulum, and vacuoles. GSH is also present in the apoplast [15]. The role and function of AsA or GSH in scavenging excess ROS produced by environmental stress have been extensively studied. Akram et al. [16] summarized the role of AsA in reducing cellular oxidative stress in detail, and these studies mentioned evaluated the effects of AsA application in several plants under various environmental abiotic stresses, including high and low temperatures [17, 18], salinity [19], and drought [20]. Exogenous GSH treatment of postharvest bell pepper alleviated injury due to chills by regulating the AsA-GSH cycle [21]. For heavy metal stresses, Chao et al. [22] reported that hydroponic AsA treatment reduces the production of malondialdehyde (MDA) and increases chlorophyll content, helping alleviate the effects of Cd poisoning in rice. In Arabidopsis, exogenous GSH treatment inhibits Cd translocation from roots to shoots, whereas endogenous GSH does not inhibit translocation [23]. Cucumber seeds treated with exogenous AsA, proline, and GSH in turn reduced Cd toxicity in seedlings [24]. In plants, APX and GSH do not act separately. AsA, which serves as a special electron donor for APX to reduce H_2_O_2_ and O_2_^−^ content, is oxidized to dehydroascorbic acid (DHAA), and the oxidized form is non-enzymatically reduced by GSH, thus forming the AsA-GSH cycle [25]. However, there is not enough research done on the interaction effects of GSH and AsA to improve environmental stress resistance, especially heavy metals in plants. Whether the ROS burst that occurs when plants encounter environmental stress induces a decrease or increase in AsA and GSH synchronously or triggers an imbalance in the AsA-GSH cycle remains unknown. These questions need to be further studied.

Currently, studies on Cd accumulation and tolerance mechanisms have mainly focused on model plants, such as Arabidopsis and rice. Extending the scope to other food crops, such as wheat, is desired. Wheat (*Triticum aestivum* L*.*) is a predominant cereal crop and the main staple food for more than 50% of the world’s population [26]. In some areas, wheat and its products are vital contributors to dietary Cd intake by people, especially in countries lacking water, where farmers use wastewater to irrigate wheat, and in areas with high soil Cd concentrations [27, 28]. Cd is more toxic to wheat than other heavy metals, such as chromium [29]. Cd hinders wheat shoot growth, causes chlorosis and necrosis of leaves, decreases leaf number and leaf area, and decreases yield [30–32]. As the major perception and adsorption site of Cd, the growth of root was also severely affected [30–32]. In this study, we selected two wheat genotypes differing in root growth under Cd toxicity. Using these genotypes, we aimed to investigate r ROS generation and the role of enzymatic and non-enzymatic antioxidant systems in countering oxidative stress generated due to Cd toxicity. These findings may provide new insight into improving wheat resistance through modulation of ROS homeostasis in Cd-contaminated areas.

## Results

### Growth performance of wheat seedlings under Cd toxicity

**Fig. 1:**
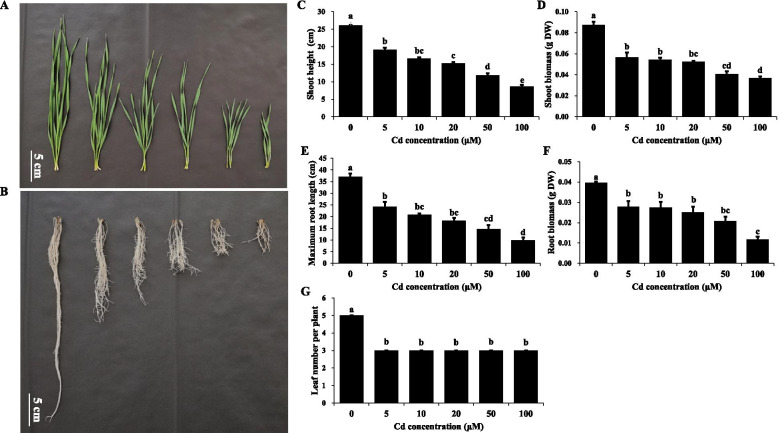
Characterization of wheat growing under different Cd concentrations. **A-B**, Images of wheat shoots (**A**) and roots (**B**) growing under different Cd concentrations for 12 d. From left to right, represent the phenotypes of plants grow in 0 μM (control), 5 μM, 10 μM, 20 μM, 50 μM, and 100 μM Cd^2+^. **C** Shoot height of wheat growing under different Cd concentrations. **D** Shoot biomass of wheat growing under different Cd concentrations. E, Maximum root length of wheat growing under different Cd concentrations. F, Root biomass of wheat growing under different Cd concentrations. G, Leaf number of wheat growing under different Cd concentrations. Results are means ± SE of six biological replicates

Increasing the Cd concentration, from 0 to 100 μM, administered to the plants caused the shoot height, root length, and the biomasses of both shoots and roots to decrease gradually compared with the control group (Fig. 1A–F). At all Cd concentrations administered inCd toxicity conditions for 12 days, the leaf number per plant was three, and the Cd-exposed wheat pants had two fewer leaves than those in Cd-free condition (Fig. 1G). As 5 μM Cd caused significant adverse effects on wheat, we selected 5 μM Cd for inducing Cd toxicity in this experiment.

**Fig. 2:**
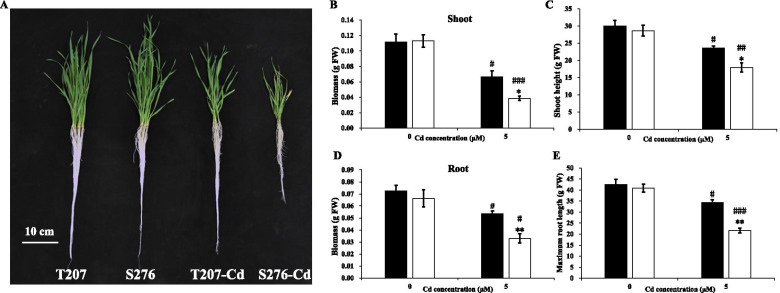
Characterization of wheat genotypes T207 and S276 growing under normal or Cd treatment. **A** Images of T207 and S276 growing under normal or Cd treatment for 12 d. **B** Shoot biomass of genotypes T207 and S276. **C** Shoot height of genotypes T207 and S276. **D** Root biomass of genotypes T207 and S276. **E** Maximum root length of genotypes T207 and S276. Results are means ± SE of six biological replicates. The black column represents T207 and the white column represents S276. The symbols # and * indicate statistically significant differences between treatments (normal and Cd conditions) (#, *P* < 0.05, ##, *P* < 0.01 and ###, *P* < 0.001) and between genotypes (T207 and S276) (*, *P* < 0.05, **, *P* < 0.01 and ***, *P* < 0.001), respectively. FW, fresh weight

Further, we investigated the physiological responses of different wheat genotypes to Cd toxicity. Cd toxicity decreased the shoot biomass up to 59.4 and 34.2%, and reduced the shoot height to 78.8 and 62.7% in the Cd-resistant genotype T207 and the Cd-sensitive genotype S276, respectively, compared with the control (Fig. 2B and C). Similarly, Cd treatment decreased the root biomass of T207 and S276 up to 73.8 and 49.7%, respectively, compared with the control (Fig. 2D). Cd toxicity also decreased the maximum root length of T207 and S276 up to 80.7 and 53.0%, respectively (Fig. 2E). The shoot height and biomass and root length and biomass were all significantly higher in T207 than S276, under Cd treatment (Fig. 2B–E). These results suggested that the wheat root, highly sensitive to Cd toxicity, could be used as an assessment of Cd resistance, and the resistance index of T207 was higher than that of S276.

### Lipid peroxidation and ROS generation in responses to Cd toxicity

**Fig. 3:**
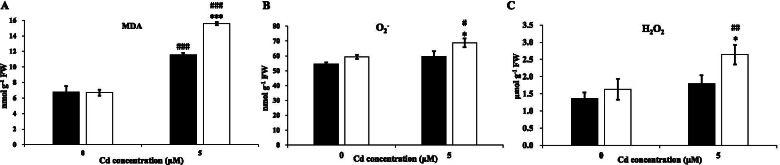
MDA, O_2_^−^, and H_2_O_2_ concentrations of wheat genotypes T207 and S276 roots growing under normal or Cd treatment. **A** MDA concentration of genotypes T207 and S276. **B** O_2_^−^ concentration of genotypes T207 and S276. **C** H_2_O_2_ concentrations of genotypes T207 and S276. Results are means ± SE of four biological replicates. The black column represents T207 and the white column represents S276. The symbols # and * indicate statistically significant differences between treatments (normal and Cd conditions) (#, *P* < 0.05, ##, *P* < 0.01 and ###, *P* < 0.001) and between genotypes (T207 and S276) (*, *P* < 0.05, **, *P* < 0.01 and ***, *P* < 0.001), respectively. FW, fresh weight

After Cd stress, the concentration of MDA, an indicator of lipid peroxidation, was evaluated in the roots of the two wheat genotypes (Fig. 3A). MDA levels were significantly increased following Cd toxicity. Under conditions of Cd toxicity, the MDA level of S276 was 35% higher than that of T207. In addition, excessive O_2_^−^ and H_2_O_2_ production in the root was found to be markedly increased in S276 after 3 days of Cd toxicity, especially the H_2_O_2_ concentration (Fig. 3B and C). The H_2_O_2_ and O_2_^−^ concentrations in the roots of S276 were 47 and 15% higher, respectively, than those in the roots of T207 under Cd toxicity. These data indicated that distinct oxidative responses may be an important contributor to the phenotypic differences between Cd-resistant T207 and the Cd-sensitive S276 genotypes in conditions of Cd stress.

### Antioxidative enzyme activities and antioxidant levels under Cd stress

**Fig. 4:**
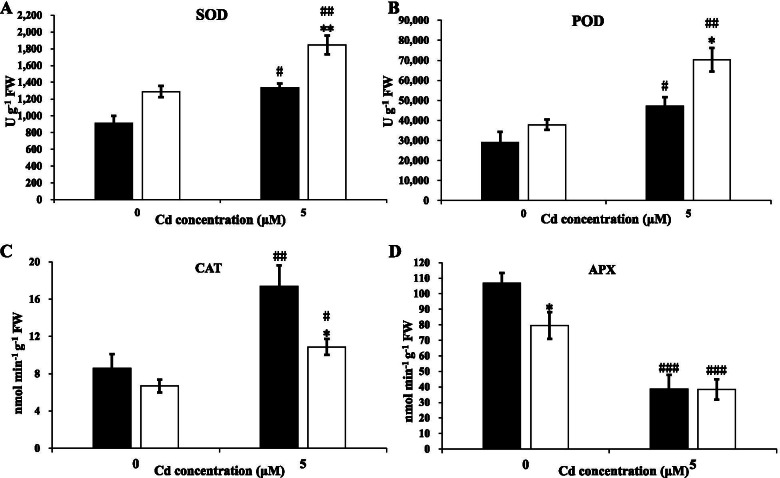
SOD, POD, CAT, and APX activities of wheat genotypes T207 and S276 roots growing under normal or Cd treatment. **A** SOD activities of genotypes T207 and S276. **B** POD activities of genotypes T207 and S276. **C** CAT activities of genotypes T207 and S276. **D** APX activities of genotypes T207 and S276. Results are means ± SE of four biological replicates. The black column represents T207 and the white column represents S276. The symbols # and * indicate statistically significant differences between treatments (normal and Cd conditions) (#, *P* < 0.05, ##, *P* < 0.01 and ###, *P* < 0.001) and between genotypes (T207 and S276) (*, *P* < 0.05, **, *P* < 0.01 and ***, *P* < 0.001), respectively. FW, fresh weight

**Fig. 5:**
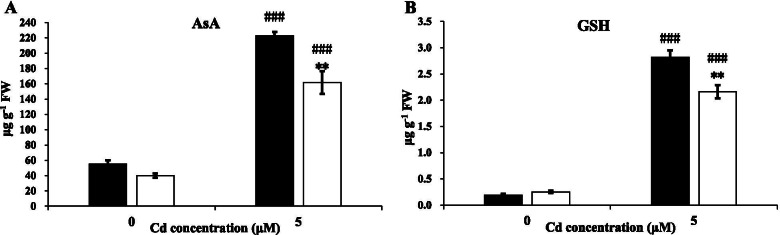
AsA and GSH concentrations of wheat genotypes T207 and S276 roots growing under normal or Cd treatment. **A** AsA concentration of T207 and S276. **B** GSH concentration of genotypes T207 and S276. Results are means ± SE of four biological replicates. The black column represents T207 and the white column represents S276. The symbols # and * indicate statistically significant differences between treatments (normal and Cd conditions) (#, *P* < 0.05, ##, *P* < 0.01 and ###, *P* < 0.001) and between genotypes (T207 and S276) (*, *P* < 0.05, **, *P* < 0.01 and ***, *P* < 0.001), respectively. FW, fresh weight

Subsequently, we examined the activities of several key antioxidant enzymes and concentrations of antioxidants in T207 and S276 genotypes. The activities of SOD, POD, and CAT were significantly increased under Cd toxicity, whereas the opposite trend was observed for APX (Fig. 4). Under Cd toxicity, the CAT activity was significantly higher in T207 than in the S276 genotype (Fig. 4B); however, the SOD and POD activities were significantly higher in S276 than in T207 (Fig. 4A, C). The APX activity showed no significant differences between S276 and T207 (Fig. 4D). The concentrations of the non-enzymatic antioxidant AsA were more than three-fold higher than those of the control after Cd toxicity in T207 and S276, respectively (Fig. 5A); similarly, for the antioxidant GSH, the levels were 1380 and 764% higher than those in T207 and S276, respectively (Fig. 5B). These two antioxidants exhibited significantly higher concentrations in T207 than in S276 under Cd toxicity (Fig. 5).

### Digital gene expression profiling of ROS-related genes

**Fig. 6:**
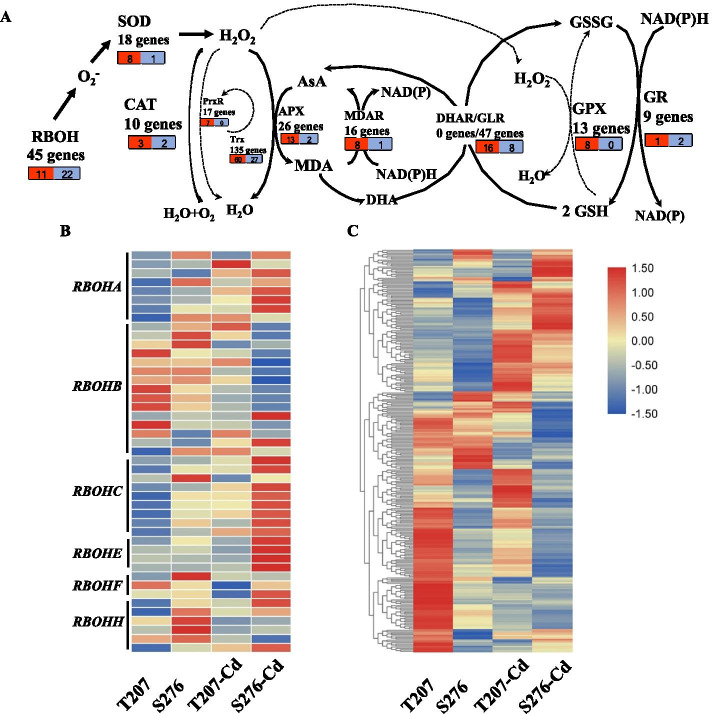
The expression of genes involved in AsA-GSH cycle. **A** Genes involved in AsA-GSH cycle in wheat root. The red box indicates the numbers of genes that showed significantly higher expression in T207 than in S276 under Cd treatment. The blue box indicates the numbers of genes that showed significantly higher expression in S276 than in T207 under Cd treatment. **B** The heatmap of RBOH expression in wheat root. **C** The heatmap of genes involved in AsA-GSH cycle expression in wheat root

The significant increase in non-enzymatic antioxidants AsA and GSH in conditions of Cd toxicity indicated that the AsA-GSH cycle might contribute significantly to Cd resistance in wheat. The AsA-GSH cycle is an important part of the ROS regulation network that includes ROS-producing and ROS-detoxifying proteins in the cell. Respiratory burst oxidase homologs, namely *RBOHs* (RBOHA-J), are mainly responsible for ROS production in plants. The ROS-detoxifying genes mainly include genes encoding iron (Fe)-, manganese (Mn)-, and copper-zinc (CuZn)-SODs, CATs, APXs, glutathione peroxidases (GPXs), and peroxiredoxins (PrxR). We investigated the expression of genes involved in the AsA-GSH cycle by high-throughput RNA-sequencing. A total of 336 genes were identified to be involved in the AsA-GSH cycle in allohexaploid wheat. In general, a higher percentage of genes had higher transcript levels in T207 than in S276 in the presence of Cd toxicity (Fig. 6A). The expression of *RBOHA*, *RBOHC*, and *RBOHE* was significantly increased, whereas that of *RBOHB* was significantly decreased by Cd toxicity (Fig. 6B). Except the *RBOHB* subfamily, almost all other genes had higher transcript abundances in T207 than in S276. A total of 22 *RBOH* homologs showed more abundant transcripts in S276 than in T207, whereas another 11 showed the opposite trend (Fig. 6A). Among the 18 *SOD* genes, the three *SOD-4A*s in wheat showed the highest expression levels and were significantly induced by Cd toxicity (Additional file 1).

Under Cd toxicity, eight genes exhibited significantly higher expression and one showed significantly lower expression in T207 than in S276. Among the 10 *CAT* genes, the expression of *CAT2* and *CATA* showed the greatest level of induction in the presence of Cd toxicity. Further, the expression of three and two *CAT* gene s was significantly higher and lower in T207 than in S276 genotypes, respectively. Among the 26 *APX* genes in wheat, the expression of 13 and two were significantly higher and lower in T207 than in S276 genotypes under Cd toxicity, respectively. Among the 16 *MDAR* genes in wheat, eight and one showed significantly higher expression and lower expression in T207 than in S276 genotypes under Cd treatment, respectively. For the 13 *GPX* genes in wheat, eight were significantly highly expressed in T207 than in S276 under Cd treatment. Among the nine *GR* genes in wheat, one exhibited significantly higher expression and two showed significantly lower expression in T207 than in S276 under Cd stress. The other genes involved in the AsA-GSH cycle included *PrxR*, *Trx*, and *GLR*. Most of these genes showed higher expressions in T207 than in S276 (Fig. 6A, C; Additional file 1). Besides, we also analyzed the genes encoding other ROS-producing enzymes such as oxalate oxidases; and proteins such as ferritin that store labile iron (Fe^2+^) to protect cell from ROS damage. For the 23 genes encoding oxalate oxidases, six and eight were significantly higher and lower in T207 than in S276 under Cd toxicity, respectively. For the five *Ferritin* genes, no significant difference was observed between the T207 and S276 genotypes under Cd toxicity.

### Root growth, lipid peroxidation, and ROS levels in response to exogenous AsA and GSH

**Fig. 7:**
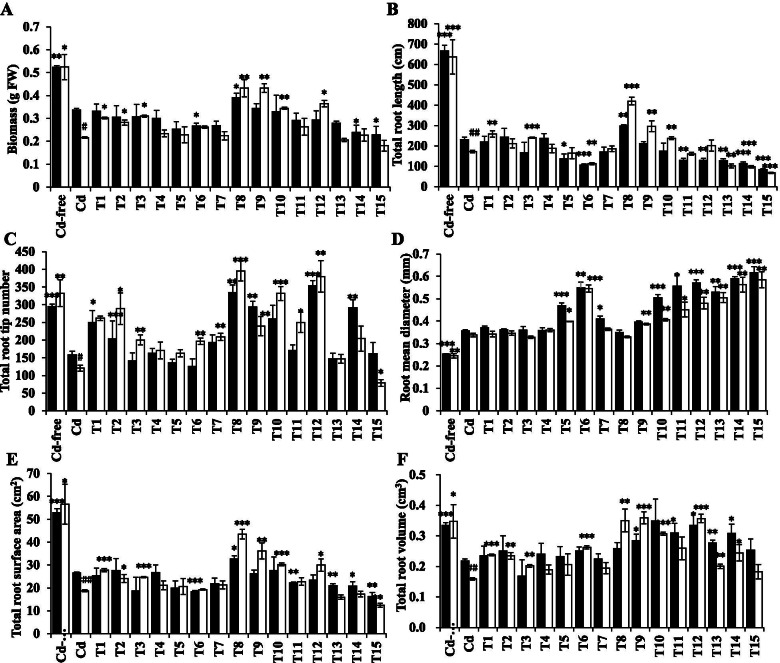
Root architecture analysis of wheat genotypes T207 and S276. Root biomass (**A**), total root length (**B**), total root tips number (**C**), root mean diameter (**D**), total root surface area (**E**), total root volume (**F**) of genotypes T207 and S276. Results are means ± SE of four biological replicates. The black column represents T207 and the white column represents S276. The symbols # indicates statistically significant differences between genotypes (T207 and S276) under Cd treatment and * indicate statistically significant differences between treatments (Cd-free and T1–15 to Cd treatment) (#, *P* < 0.05, ##, *P* < 0.01 and ###, *P* < 0.001), respectively. FW, fresh weight

**Fig. 8:**
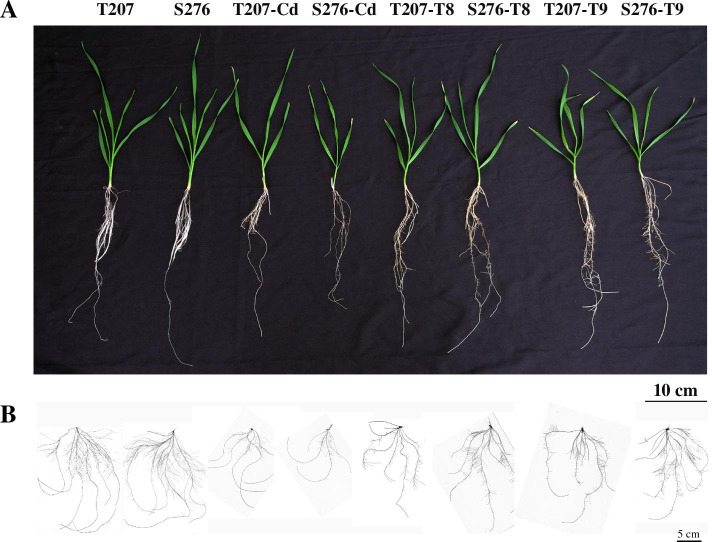
The phenotypes of wheat genotypes T207 and S276 in response to exogenous AsA and GSH. **A** Whole plant phenotypes. **B** Root phenotypes. Cd: 5 μM Cd^2+^, T8: 50 μM GSH + 0.1 mM AsA + 5 μM Cd^2+^, T9: 100 μM GSH + 0.1 mM AsA + 5 μM Cd^2+^

Based on the results obtained, Cd stress increased the synthesis and accumulation of the antioxidants AsA and GSH. Therefore, we investigated the roles of these two antioxidants in reducing Cd toxicity-mediated oxidative damage. GSH application alone (T1-T3) significantly increased root biomass, total root length, root tip number, total root surface, and total root volume of S276 (Fig. 7A–C, E, F). AsA application alone (T4-T6) had relatively weaker effects on root system architecture (RSA), and it decreased the total root length and increased the root mean diameter significantly at a high concentration (T6: 0.8 mM AsA) (Fig. 7B, D), which usually indicates more severe Cd toxicity. The combined use of AsA and GSH significantly changed RSA. Application of 50 μM GSH + 0.1 mM AsA (T8) to Cd-stressed wheat significantly increased the root biomass, total root length, root tip number, total root surface, and total root volume (Fig. 8A–C, E, F). Additionally, the mean root diameter did not change (Fig. 7D). Therefore, T8 (50 μM GSH + 0.1 mM AsA) was the best combination among the nine combinations evaluated. T9 (100 μM GSH + 0.1 mM AsA) treatment had similar effects to T8. The effect of improving wheat growth is mainly reflected in the genotype S276, which exhibits a greater vulnerability to Cd toxicity. Figure 8 shows the phenotype of Cd-free, 5 μM Cd, 5 μM Cd + T8 (50 μM GSH + 0.1 mM AsA), and 5 μM Cd + T9 (100 μM GSH + 0.1 mM AsA)-treated wheat under Cd stress. Shoot and root growth were improved by the two treatments.

**Fig. 9:**
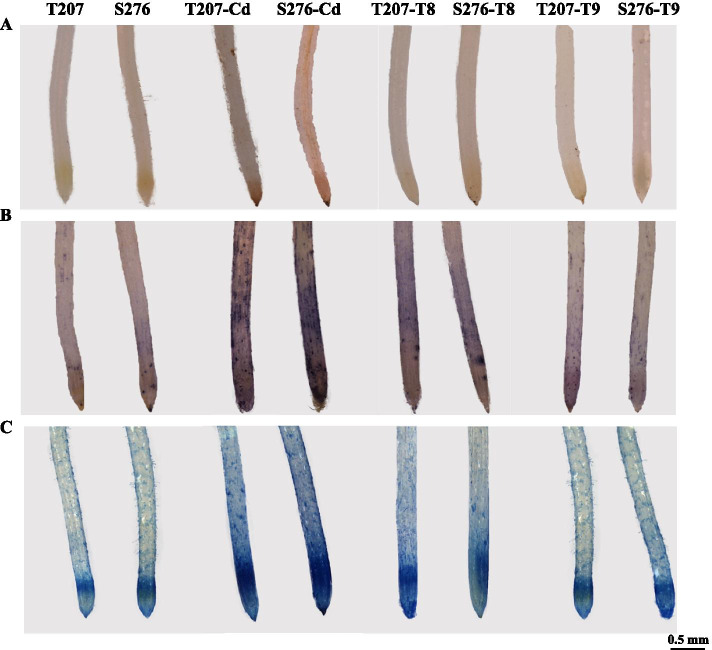
Histochemical detection of H_2_O_2_, O_2_^−^, and cell death of wheat genotypes T207 and S276 in response to exogenous AsA and GSH. **A** DAB staining of roots. **B** NBT staining of roots. **C** Evan’s blue staining of roots. Cd: 5 μM Cd^2+^, T8: 50 μM GSH + 0.1 mM AsA + 5 μM Cd^2+^, T9: 100 μM GSH + 0.1 mM AsA + 5 μM Cd^2+^

Further analysis after 3,3-N-diaminobenzidine tetrahydrochloride (DAB), nitroblue tetrazolium chloride (NBT), and Evans blue staining were performed to identify the H_2_O_2_ and O_2_^−^ levels and cell viability, respectively. For the above staining methods, the roots were stained stronger after induction of Cd toxicity in both genotypes, especially in S276 (Fig. 9), which was consistent with the ROS and MDA levels (Fig. 3). The exogenous application of 50 μM GSH + 0.1 mM AsA and 100 μM GSH + 0.1 mM AsA significantly decreased DAB, NBT, and Evans blue staining levels, especially in the S276 genotype (Fig. 9).

## Discussion

Numerous studies have reported that Cd-contaminated soil adversely affects all aspects of plants. Root growth inhibition is a primary symptom of Cd toxicity and can be used to establish phenotypic and genotypic differences for Cd sensitivity or tolerance [33]. In this study, two wheat genotypes with differential root growth responses to Cd resistance were selected. Subsequently, a series of physiological and transcriptomic analyses were conducted to identify the mechanisms underlying the contrasting Cdresistance observed.

Physiological and biochemical studies indicated that Cd administration induced considerable ROS accumulation in the roots. Lower levels of ROS were produced in the Cd-resistant genotype T207 than in the Cd-sensitive genotype S276 (Figs. 3 and 9). Further, the MDA content and intensity of Evans blue staining, indicators of plasma membrane damage and cell death, were consistently higher in S276 than in T207 (Figs. 3 and 9), which might explain the Cd inhibition of root elongation in S276. RBOHs are the primary source of plasma membrane-associated ROS [34]. In the Arabidopsis genome, 10 *RBOH* homologous genes (*AtRBOH A-J*) have been annotated [35]. Different *RBOH* copy numbers exist in other plant species, including six in barley [36] and 20 in *Nicotiana tabacum* [37]. Differentially expressed in different plant tissues, *RBOH*-mediated ROS burst significantly affects plant physiological processes such as pollen tube growth and root hair cell expansion; and plant defenses against biotic and abiotic stresses, including heavy metals [38, 39]. identified The NADPH oxidase (RBOH) was identified to be a main source of Cd-induced H_2_O_2_ using diphenylene iodonium, an oxidase inhibitor [40]. However, the authors did not identify which *RBOH* gene was the main contributor. In Arabidopsis, Cd administration induced the expression of *RBOHC* and *RBOHD* in leaves and *RBOHF* in roots exposed to low Cd concentrations [41]. Using the Arabidopsis mutants *rbohC*, *rbohD*, and *rbohF*, Gupta et al. [38] found that compared with the wild type, the roots of these mutants grew poorly, whereas their leaves showed improved growth after 5 days of Cd toxicity. These studies showed that different *RBOH* genes differentially regulate ROS metabolism under Cd toxicity conditions. Among the 45 *RBOH* genes in wheat, in general, the homologs of *RBOHA*, *RBOHC*, and *RBOHE* were significantly induced by Cd toicity, and the expression levels of 22 *RBOH* genes were higher in the S276 than T207 genotype, which was consistent with the higher Cd-induced ROS content in S276 than T207 (Fig. 3B, C). Interestingly, *RBOHB* expression was significantly decreased by Cd toxicity, and its expression level was higher in T207 than in S276 (Fig. 6; Additional file 1). RBOHB is a root-specific protein in Arabidopsis [42] and functions in lateral root development in *Phaseolus vulgaris* [43]. Here, we found that root tip numbers were significantly decreased by administration of Cd toxicity, especially in the S276 genotype (Fig. 7C), which may be related to the decreased expression of *RBOHB*. This phenomenon of the contrasting function of RBOHB compared with RBOHD and RBOHF has also been reported in *Arabidopsis* resistance against nematodes [42]. These results indicate that there is an intricate and finely tuned transcriptional regulation mechanism that restricts ROS production under Cd stress in wheat.

To minimize the ROS-induced oxidative damage, plants have evolved a sophisticated defense antioxidative system consisting of enzymatic and non-enzymatic components. Among the antioxidative enzymes, SOD and POD are the first line of defense against ROS. SOD catalyzes the dismutation of O_2_^−^ to H_2_O_2_ and molecular oxygen [12], whereas CAT and APX subsequently convert H_2_O_2_ to H_2_O [44]. A previous study showed that Cd is a metal without redox-active properties, which therefore did not participate directly in Fenton-type reactions—the source of ROS [45]. Therefore, Cd may cause the indirect accumulation of ROS by inhibiting antioxidant enzymes. However, a few studies have shown that variations in antioxidative enzyme activity under oxidative stress can be contradictory: increase, decrease, or no change can be observed [46, 47]; this is attributable to analysis in different plant species, plant tissues, or durations and concentrations of metal exposure. In this study, except for APX, activities of SOD, POD, and CAT were significantly induced by Cd (Fig. 4). This may be due to increased ROS production. Between the two genotypes, SOD and POD activities were significantly higher, whereas the CAT activity was significantly lower in the Cd-sensitive S276 than in the Cd-resistant T207 genotype. The basal levels of these enzymes showed similar trends (Fig. 4). These results were similar to those for different wheat genotypes in response to aluminum (Al) toxicity [48]. This suggests common mechanisms underlying wheat resistance to Cd and Al stress. Among the *APX* genes in Arabidopsis, cytosolic *APX1* is a central component of the ROS gene network [49]. In this study, *APX1* in wheat was significantly upregulated by Cd toxicity. However, the APX activity was significantly decreased, which is consistent with the response of wheat to copper toxicity [50], suggesting post-transcriptional regulation of the APX activity in wheat. The AsA-GSH cycle — an important pathway to control H_2_O_2_ levels in cells — is present in all cell compartments. AsA and GSH react directly with H_2_O_2_ to eliminate its toxicity, and the regeneration of AsA requires GSH as a reductant [44]. In this study, AsA and GSH concentrations were increased greatly on exposure to Cd (Fig. 5). Similar results have been reported in Arabidopsis [38]. AsA and GSH concentrations were significantly higher in T207 than in S276 in the presence of Cd toxicity (Fig. 5), which also disturbed the expression of genes involved in the AsA-GSH cycle. A greater number of upregulated genes were identified in the AsA-GSH cycle in T207 than in S276 (Figs. 4, 5 and 6). These observations indicated that the AsA-GSH cycle in T207 was more efficient than that in S276 under Cd stress. With the development of molecular biology and genetic engineering (including metabolomics, epigenomics, CRISPR-Cas9, and high-throughput sequencing technologies), the identification of target genes that may help improve crop abiotic stress tolerance has been possible. Zafar et al. [51] have proposed that targeting ROS-related genes can achieve abiotic stress tolerance; for example, plants overexpressing melatonin (an antioxidative molecule that helps plants scavenge ROS) biosynthesis genes were found to be tolerant to different abiotic stresses. As a hexaploid crop, wheat contains a large number of repetitive sequences and homologous chromosome segments, and hence multi- copies gene familiesin its genome. Using RNA-seq and comparisons between Cd toxicity tolerant and sensitive genotypes, we identified two *APX1* copies (*TraesCS4A02G106300* and *TraesCS4D02G198500*) that showed high expression levels, were strongly induced by Cd toxicity, and had significantly higher abundance in the Cd toxicity tolerant genotype than in the sensitive genotype (Additional file 1). These two genes may be promising gene modification targets that may help promote wheat Cd tolerance through maintaining redox balance in cells.

Both exogenous AsA and GSH can increase cell division activity and influence cell differentiation in the root apical meristem of Arabidopsis [52, 53]. However, their application does not result in the same effects on the RSA. AsA treatment promotes root length [52], whereas GSH promotes both the number and length of lateral roots [54]. Recent studies have demonstrated that the exogenous application of AsA or GSH can alleviate heavy metal stress. For example, Jung et al. found that exogenous AsA [55] or GSH [56] application alleviated arsenic toxicity in rice seedlings. Alamri et al. found that AsA and GSH independently alleviated arsenate toxicity in brinjal [57]. The role of AsA and GSH in alleviating Cd toxicity in wheat remains unclear. Cd significantly hinders plant root growth, reduces root length, increases root diameter [58], and inhibits root branching [59]. In this study, under Cd toxicity, exogenous administration GSH increased total root numbers, increasing the total root surface, total root volume, and root biomass. Exogenous ASA increased root mean diameter, decreased root length and diameter, and had no significant effect on the root surface, total root volume, and root biomass (Fig. 7). A previous study showed that spraying ASA alone alleviated Cd-induced oxidative stress in rapeseed plants, whereas the aboveground biomass alone was significantly affected [60]. The result is similar to that from our study showing that the effect of AsA on the root biomass was slight (Fig. 7A), and high ASA resulted in shorter and thicker roots (Fig. 7B, D). Although AsA application alone did not improve the architecture of Cd-treated roots, the combined application of AsA and GSH significantly relieved the adverse growth of roots induced by Cd, with significantly better results obtained than with GSH addition alone (Fig. 7). The combined application of AsA and GSH to Cd-stressed wheat seedlings increased root biomass, total root length, and total root tip numbers, and decreased the levels of MDA, O_2_^−^, and H_2_O_2_ compared to those under Cd toxicity (Figs. 7 and 9). Our results showed that Cd-induced root growth impairment in wheat was correlated with the AsA and GSH levels. Plants may increase the activity of the AsA-GSH cycle to overcome Cd toxicity (Fig. 4). AsA and GSH contribute significantly to ROS scavenging and redox regulation under heavy metal stress [22, 61, 62] have been extensively studied. Their combined effects were studied here for the first time, and a 0.1 mM AsA + 50 μΜ GSH was found to alleviate Cd toxicity most significantly. Integrating the above theoretical analyses and experimental results, the combination application of exogenous AsA and GSH to Cd-exposed wheat, enhanced the metabolic intensity of the AsA-GSH cycle to scavenge excess ROS, and fine-tuned the root growth synergistically, alleviating Cd toxicity. This study highlighted the pivotal role of ROS homeostasis in determining Cd resistance in wheat genotypes through integrated physiological and transcriptional analyses. In the near future, the core genes regulating ROS production and scavenging should be identified in allohexaploid wheat, and characterization of the corresponding favorable alleles in the Cd-resistant wheat genotypes may provide elite gene resources for the genetic improvement of wheat Cd resistance.

## Conclusions

To understanding the Cd resistance mechanisms in wheat, two wheat genotypes, the Cd-resistant T207 and the Cd-sensitive S276 were selected and the physiological and transcriptomic analysis were conducted.The results showed that the differential production of ROS by Cd had a key role in wheat Cd resistance. The Cd resistant genotypeT207 showed a better root architecture system, lower O_2_^−^, H_2_O_2_, and MDA concentrations, higher CAT activity, and more abundant AsA and GSH levels than the Cd-sensitive genotype S276 under Cd toxicity. Root transcriptomic profiling for T207 and S276 indicated that Cd administration-induced the transcriptional rearrangement of root genes involved in the ROS burst and the AsA-GSH cycle. Overall, the *RBOH* homologs were more highly expressed in S276 than in T207 and a more active ROS-scavenging AsA-GSH cycle was identified in T207 than in S276. Our results also demonstrate that exogenous application of AsA and GSH alleviates Cd toxicity in wheat plants by scavenging excess ROS and regulating root growth synergistically, especially in the Cd-sensitive genotype. The combination of 0.1 mM AsA with 50 μΜ GSH showed significant detoxification effects.

The results reported here provide valuable insight into the critical role of the ROS-mediated differential Cd sensitivity and the AsA-GSH cycle-mediated Cd resistance in wheat. . Two *APX1* copies (*TraesCS4A02G106300* and *TraesCS4D02G198500*) were identified as key players in the Cd toxicity. However, no further functional verification of these two genes was performed. Further genetic analysis of the elite genes is required to understand the Cd-detoxifying mechanisms and develop safer food in Cd-contaminated soils.

## Methods

### Plant material

In this study, wheat seeds were provided by the Wheat Research Institute, Henan Academy of Agricultural Sciences. Among the 126 wheat genotypes, we selected accession number 207 as a Cd toxicity-resistant genotype, and accession number 276 as a Cd toxicity-sensitive genotype. We named them T207 and S276, respectively, in this study.

### Plant growth

Hydroponic culture was used for seed germination and seedling growth. Plump wheat seeds were surface-sterilized using 0.5% (w/v) NaClO for 10 min and subsequently rinsed completely with pure water. After being allowed to germinate on gauze for 5 days, uniform seedlings were transplanted into black plastic containers with Hoagland and Arnon [63] solution. The full-strength solution contained 5.0 mM KNO_3_, 5.0 mM Ca(NO_3_)_2_, 2.0 mM MgSO_4_·7H_2_O, 1.0 mM KH_2_PO_4_, 50 μM EDTA-Fe, 46 μM H_3_BO_3_, 9.0 μM MnCl_2_·4H_2_O, 0.80 μM ZnSO_4_·7H_2_O, 0.37 μM Na_2_MoO_4_·2H_2_O, and 0.30 μM CuSO_4_·5H_2_O. The nutrient solution was replaced every 3 days. The wheat seedlings were grown in one-quarter-strength, one-half-strength, and eventually full-strength solutions. The plants were grown in a climate chamber using a temperature setting of 24/22 °C (day/night), a photoperiod of 14/10 h (day/night), and light intensity of 300–320 μmol m^− 2^ s^− 1^.

### Experimental design Cd concentration gradient treatments and selection of tolerant and sensitive cultivars

Uniform wheat seedlings after 5-d seed germination were treated with CdCl_2_ at six concentrations: 0, 5, 10, 20, 50, and 100 μM for 12 days. Shoot height (cm) and biomass (g), root length (cm) and biomass (g), and leaf number were recorded.

A total of 126 wheat genotypes germinated and grew in pure water for 5 days, and the seedlings were transferred to a nutrient solution containing 5 μM Cd^2+^. Root biomass and length were measured after 12 days. The genotype with extremely high and low root biomass and length was selected as Cd-tolerant and sensitive genotypes, respectively.

### Physiological and transcriptional responses to Cd^2+^ in both cultivars

Uniform wheat seedlings after 5-d seed germination were grown in normal solution for 7 days, and were divided into two experimental groups based on Cd-free (normal culture) and Cd (5 μM Cd^2+^) treatments. After 3 days, the samples were harvested to evaluate ROS concentrations, antioxidative enzyme activity, antioxidant concentrations, and for transcriptome sequencing.

### Addition of exogenous antioxidants

Uniform wheat seedlings after 5-d seed germination were grown in normal solution for 7 days and were subsequently treated with differential exogenous antioxidant concentrations. The treatment plan is outlined in Table 1. After 7 days, the RSA parameters were analyzed and root microscopy was performed.

**Table 1:** Addition of exogenous antioxidants for different treatments

Treatment	*Cd-free*	*Cd*	*T1*	*T2*	*T3*	*T4*	*T5*	*T6*	*T7*	*T8*	*T9*	*T10*	*T11*	*T12*	*T13*	*T14*	*T15*
Cd^2+^ (μM)	0	5	5	5	5	5	5	5	5	5	5	5	5	5	5	5	5
GSH (μM))	0	0	20	50	100	0	0	0	20	50	100	20	50	100	20	50	100
AsA (mM)	0	0	0	0	0	0.1	0.4	0.8	0.1	0.1	0.1	0.4	0.4	0.4	0.8	0.8	0.8

### Evaluation of ROS, MDA, antioxidant concentrations, and antioxidative enzyme activities

Root samples were homogenized in 50 mM PBS buffer (pH = 7.8). The homogenates were centrifuged at 8000×*g* for 15 min at 4 °C. The supernatant was treated with 10 mM hydroxylamine hydrochloride and bathed in warm water at 25 °C for 1 h. Next, 17 mM p-aminobenzene sulfonic acid and 7 mM α-naphthylamin were added to the reaction solution and placed in warm water at 25 °C for 20 min. The O_2_^−^ concentration was analyzed spectrophotometrically at 530 nm. For H_2_O_2_ level analysis, root samples were homogenized using precooled acetone and centrifuged at 10000×*g* for 10 min at 4 °C. The supernatant was reacted with 20% titanium chloride. After mixing well, 40 μl of concentrated ammonia was added for sample precipitation. The precipitate was washed three times with acetone and then dissolved in 1 M H_2_SO_4_. The absorbance at 410 nm was measured.

The level of lipid peroxidation is usually indicated by the MDA content [64]. Root samples were homogenized in 5% trichloroacetic acid. The homogenates were centrifuged at 3000×*g* for 10 min at 4 °C. The supernatant was reacted with 0.67% thiobarbituric acid (TBA) and placed in boiling water for 30 min to form a red product. The homogenates were centrifuged at 10000×*g* for 10 min at room temperature. The supernatant was assayed spectrophotometrically at 450 nm, 532 nm, and 600 nm. The MDA concentration was calculated as follows: C/μmol/L = 6.45*(A_532_-A_600_)-0.56*A_450_ [64].

For antioxidative enzyme activity analysis, root samples were homogenized in 50-mM phosphate buffer (PBS, pH = 7.0) containing 1 μM EDTA. The homogenates were centrifuged at 14000×g for 20 min at 4 °C. Subsequently, the supernatant was used for the enzyme assays based on the method of Zafar et al. [65]. O_2_^−^ reduces NBT to produce formazan. SOD has anti-oxidant activity against O_2_^−^; thus formazan content indicates SOD activity [66]. One unit (U) of SOD activity represents 50% inhibition of formazan production. POD catalyzes H_2_O_2_ and forms guaiacol via dehydrogenation. POD activity was monitored by the formation of guaiacol dehydrogenation at 470 nm. CAT activity was monitored by analyzing the decomposition of H_2_O_2_ at 240 nm. APX activity was determined by observing the decrease in AsAabsorbance at 290 nm over 2 min. SOD, POD, CAT, and APX activities were evaluated using the following assay kits: SOD-1-Y, POD-1-Y, CAT-1-Y, and APX-1-W, respectively (Coming Medical Technology Co., Ltd., Suzhou, China).

For antioxidant assays, AsA was analyzed by reacting the supernatant with Fast Blue B salt and the reaction was assayed spectrophotometrically at 420 nm. GSH was analyzed after reacting it with 5,5′-Dithiobis-2-nitrobenzoic acid and the reaction products were assayed spectrophotometrically at 412 nm. AsA and GSH were evaluated using the following assay kit: ASA-1-W and GSH-1-W, respectively (Coming Medical Technology Co., Ltd., Suzhou, China).

### Microscopy analysis

H_2_O_2_ was analyzed in root samples by immersion in 0.5% (w/v) DAB, and the samples were incubated at room temperature until the root turned brown [35]. O_2_^−^ was detected by reacting with 0.2% (w/v) NBT at 37 °C to produce a blue formazan precipitate in the root [67]. The loss of cell viability was analyzed using 0.25% (w/v) Evans blue solution-based staining for 5 min and the samples were washed three times with 100 μM CaCl_2_ solution (pH = 5.6) [68]. A fluorescent microscope (LSM800, Carl Zeiss, Oberkochem, Germany) was used for observation.

### High-throughput RNA-sequencing and differential expression analysis

Wheat roots were harvested from Cd-free and Cd-treated plants for transcriptomic analysis, using three biological replicates for analysis. Purified total RNA of the fresh wheat roots were sequenced using an Illumina Hiseq X Ten platform (Illumina Inc., San Diego, CA, USA). The cDNA libraries were prepared following Tru-Seq™ RNA sample preparation Kit from Illumina (Illumina Inc., San Diego, California, USA). A total of 12 RNA samples were analyzed, which generated 6.0 Gb of sequencing data with 150 bp paired-end (PE) reads per sample. The raw paired end reads were trimmed and quality controlled by SeqPrep (https://github.com/jstjohn/SeqPrep) and Sickle (https://github.com/najoshi/sickle) with default parameters. Then, clean reads were separately aligned to reference genome with orientation mode using TopHat (http://tophat.cbcb.umd.edu/, version 2.0.0) Transcript abundances (FPKM values) were calculated from RNA-seq data using the method described by Zhou et al. [69].The differentially expressed genes (DEGs) were defined as those with a *p* value and false discovery rate that were less than 0.05.

### Determination of root system architecture

WinRHIZO (Pro 2013a, Regent Instrument Inc.), a root analysis software, was used to measure the RSA parameters, including total root length, average root diameter, root tip number, root surface area, and total root volume.

### Statistical analysis

A randomized block design was used in all trials. Using GraphPad Prism 5.01, the differences among Cd concentration gradient treatments were evaluated using the Tukey test. The significant differences between control and treatment groups (Cd-free and Cd stress) or between genotypes (T207 and S276) were evaluated with the Student’s *t*-test. Differences were considered statistically significant at *p* < 0.05.

## Supplementary Information

**Additional file 1.** The expression of genes involved in AsA-GSH cycle.

## Data Availability

The datasets generated and analyzed during the current study are available from the corresponding author on reasonable request.

## Abbreviations

Cd: Cadmium
AsA: Ascorbic acid
GSH: Glutathione
ROS: Reactive oxygen species
SOD: Superoxide dismutase
POD: Peroxidase
CAT: Catalase
APX: Ascorbate peroxidase
GST: Glutathione-S-transferase
MDHAR: Monodehydroascorbate reductase
GR: Glutathione reductase
NADPH: Nicotinamide adenine dinucleotide phosphate
MDA: Malondialdehyde
DHAA: Dehydroascorbic acid
RBOH: Respiratory burst oxidase homolog
GPX: Glutathione peroxidase
PrxR: Peroxiredoxins
DPI: Diphenylene iodonium
RSA: Root system architecture
TBA: Thiobarbituric acid
PBS: Phosphate buffer
NBT: Nitroblue tetrazolium chloride
DAB: 3,3-N-diaminobenzidine tetrahydrochloride
